# Highly ordered macroporous dual-element-doped carbon from metal–organic frameworks for catalyzing oxygen reduction†

**DOI:** 10.1039/d0sc02518f

**Published:** 2020-08-11

**Authors:** Wei Xia, Michelle A. Hunter, Jiayu Wang, Guoxun Zhu, Sarah J. Warren, Yingji Zhao, Yoshio Bando, Debra J. Searles, Yusuke Yamauchi, Jing Tang

**Affiliations:** School of Chemistry and Molecular Engineering, Shanghai Key Laboratory of Green Chemistry and Chemical Processes, East China Normal University Shanghai 200062 China; Australian Institute for Bioengineering and Nanotechnology (AIBN), The University of Queensland Brisbane QLD 4072 Australia tangjing0206@sina.cn y.yamauchi@uq.edu.au d.bernhardt@uq.edu.au; International Center for Materials Nanoarchitectonics (MANA), National Institute for Materials Science (NIMS) 1-1 Namiki Tsukuba Ibaraki 305-0044 Japan; School of Chemistry and Molecular Biosciences, The University of Queensland Brisbane QLD 4072 Australia; Australian Institute for Innovative Materials (AIIM), The University of Wollongong Squires Way North Wollongong NSW 2500 Australia; Institute of Molecular Plus, Tianjin University No. 11 Building, No. 92 Weijin Road, Nankai District Tianjin 300072 P. R. China; School of Chemical Engineering, The University of Queensland Brisbane QLD 4072 Australia; Department of Plant & Environmental New Resources, Kyung Hee University 1732 Deogyeong-daero, Giheung-gu Yongin-si Gyeonggi-do 446–701 Republic of Korea

## Abstract

Multiple heteroatom-doped carbons with 3D ordered macro/meso-microporous structures have not been realized by simple carbonization of metal–organic frameworks (MOFs). Herein, ordered macroporous phosphorus- and nitrogen-doped carbon (M-PNC) is prepared successfully by carbonization of double-solvent-induced MOF/polystyrene sphere (PS) precursors accompanied with spontaneous removal of the PS template, followed by post-doping. M-PNC shows a high specific surface area of 837 m^2^ g^−1^, nitrogen doping of 3.17 at%, and phosphorus doping of 1.12 at%. Thanks to the hierarchical structure, high specific surface area, and multiple heteroatom-doping, M-PNC exhibits unusual catalytic activity as an electrocatalyst for the oxygen reduction reaction. Computational calculation reveals that the P=O group helps stabilize the adsorption of intermediates, and the position of P=O relative to graphitic N significantly improves the activity of the adjacent carbons for electrocatalysis.

## Abstract 2

Multiple heteroatoms-doped carbon with 3D ordered macroporous structures, which showing outstanding catalytic activity for oxygen reduction, was prepared by carbonization of double-solvent-induced MOF/polystyrene sphere accompanied with post-doping.

## Graphical abstract

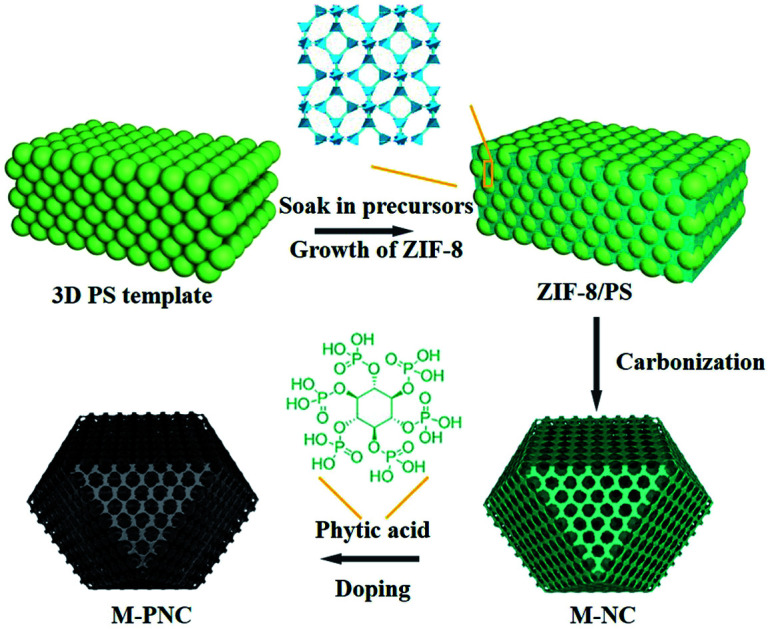

## Introduction

Nanoporous functional carbon-based materials are considered to be promising low-cost electrocatalysts in various research fields, including for oxygen reduction reaction (ORR),^1^ carbon dioxide reduction reaction (CRR),^2^ and nitrogen reduction reaction (NRR),^3^*etc.* At present, metal–organic frameworks (MOFs) have been widely applied as reasonable precursors to prepare inorganic nanomaterials (*e.g.* carbon) with controllable frame structure, high porosity, large surface area, and doped functional heteroatoms.^4^ In most cases, common MOF-derived carbons are solid polyhedrons with mainly micropores, whose morphology is inherited from the MOF parents. However, the present micropores in porous materials are prone to be barricaded in by reactants and products during complex electrocatalysis, leading to the reduction of available active sites.^5^

To provide carbons with uniform pore opening, a high specific surface area, and advanced approachability, it is of great importance to construct three-dimensional (3D) hierarchically ordered macro-meso-microporous architectures. In particular, the introduction of ordered macropores not only boosts the penetration of the electrolyte to shorten the electrolyte diffusion paths, but also enhances the structural integrity of the electrocatalysts. Furthermore, high-pressure treatment is sometimes used in the preparation of electrodes to increase volume-normalized activity. 3D macroporous structures undergoing squeeze processing still contribute to superior mass transfer in electrocatalytic reactions.^6^ Recently, Shen and co-workers^7^ first designed a single-crystalline ZIF-8 with the 3D ordering of macro–micropores *via* a double-solvent-induced nucleation method with a polystyrene sphere (PS) template. Based on the above research, hierarchical ordered porous nitrogen-doped carbon-based nanomaterials were reported by carbonization of 3D ordered macroporous ZIF single crystals, and were used as the electrode material for potassium–ion batteries^8^ or aluminium–ion batteries.^9^ However, a large amount of toxic organic solvents (*e.g.*, tetrahydrofuran and *N*,*N*-dimethylformamide) are utilized for the removal of PS templates in these studies to prepare macroporous ZIF crystals first, which results in environmental pollution, tedious processes, and a waste of solvents. The prepared 3D ordered macroporous single ZIF crystals are further converted into carbons by carbonization. It is much simpler, more economical and environmentally-friendly to realize the removal of PS and the formation of intact ordered macroporous carbon through a one-step heat-treatment process.

Also, the electrocatalytic properties of carbon-based nanomaterials not only depend on the capacity of mass/electron transportation^10^ but also on the intrinsic activity determined by the doping heteroatoms.^11^ Porous carbon nanostructures loaded with transition metals still have the limitation of agglomeration and poor distribution of metal nanoparticles.^12^ Thus, heteroatom-doped porous carbon materials with excellent activity are more inclined to be desirable Pt-alternative electrocatalysts. Recently, the modulation of carbon with heteroatoms (P, N, B, S, *etc.*) has been shown to provide a valid approach to improve the electrocatalytic activity for ORR, CRR, and NRR,^13^ due to the altered surface electronic structure of carbon and increased active sites.^14^ In the case of catalyzing the ORR, doping N atoms into a carbon nanostructure can induce a positive charge density on neighbouring carbon atoms, working as enhanced O_2_ adsorption and reduction sites.^15^ Moreover, introducing other heteroatoms into nitrogen-doped carbon nanomaterials tends to show a synergistic effect and generate abundant active sites other than N-sites, which are beneficial to boosting electrochemical activity.^16^ In particular, doped P atoms, with their lower electronegativity than N due to their 3p lone pair electrons, can fine-tune the charge and spin distribution of nitrogen-doped carbon materials,^17^ leading to the increased catalytic activity for ORR.

Herein, ordered macroporous nitrogen-doped carbon was prepared successfully by simple carbonization of double-solvent-induced ZIF-8/PS precursors which combines the carbonization of ZIF-8 and spontaneous removal of the PS template. A face-centred cubic PS template was used to grow the single-crystal ZIF-8 (ZIF-8/PS). Then, ordered macroporous nitrogen-doped carbon (M-NC) was obtained by calcinating the ZIF-8/PS precursor under an inert atmosphere, then this was post-doped with phosphorus to form ordered macroporous phosphorus and nitrogen-doped carbon (M-PNC). M-PNC prepared at 1000 °C showed a highly specific surface area of 837 m^2^ g^−1^, nitrogen doping of 3.17 at%, phosphorus doping of 1.12 at%, as well as abundant hierarchical porous structures. To prove the concept of construction of the hierarchical structure and multiple heteroatom-doping, M-PNC was tested as an electrocatalyst for ORR. Computational models of P, N doped carbon systems were also designed to investigate the intrinsic catalytic active sites in phosphorus and nitrogen-doped carbon nanomaterials.

## Results and discussion

**Scheme 1:**
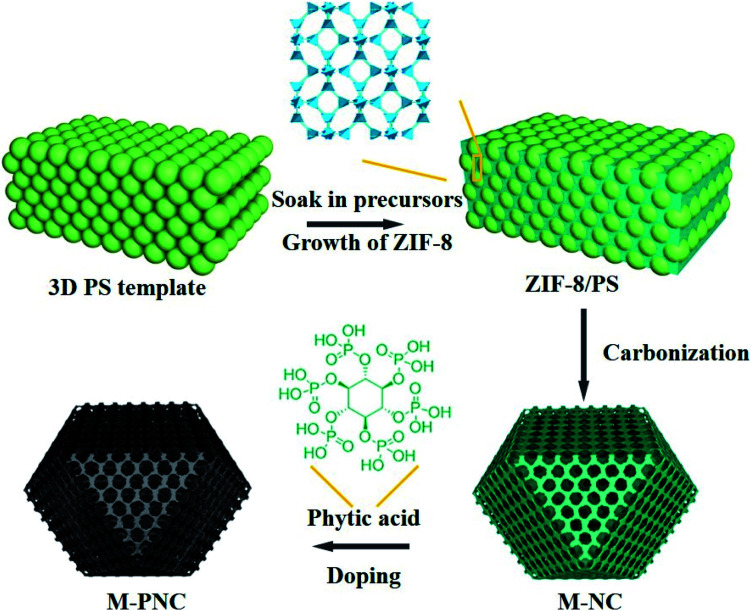
Illustration for preparing ordered macroporous phosphorus and nitrogen-doped carbon (M-PNC).

The preparation procedure of ordered macroporous phosphorus and nitrogen-doped carbon (M-PNC) is shown in Scheme 1. First, a 3D ordered polystyrene sphere (PS) template was soaked in a methanolic solution containing saturated 2-methylimidazole and zinc nitrate. Growth of the ZIF-8 crystalline phase occurs in the voids of the face-centred cubic PS template to form ZIF-8/PS, induced by replacing the solvent with a mixed ammonia-methanol solution. Afterwards, the ZIF-8/PS was calcined under an inert atmosphere to realize the simultaneous carbonization of ZIF-8 and removal of the PS template. 3D ordered macroporous nitrogen-doped carbon (M-NC) was obtained at this stage. Finally, the resultant M-NC was mixed with phytic acid to produce M-PNC *via* thermal treatment in an inert atmosphere. Importantly, the dual-doping and open hierarchical porous structure makes M-PNC an ideal electrochemical catalyst.

### Characterization of M-NC

**Fig. 1:**
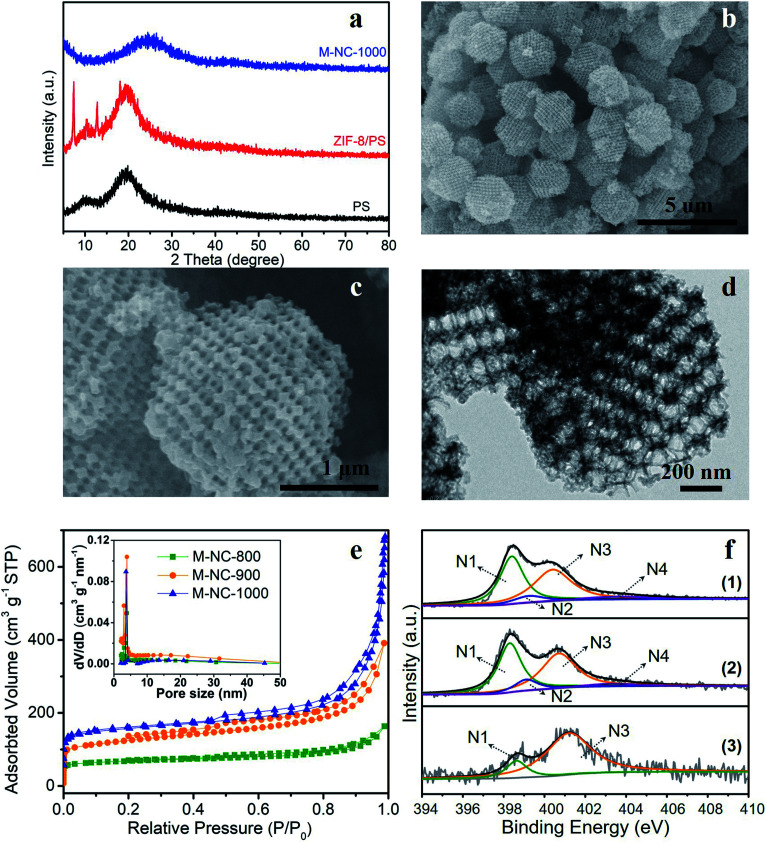
Morphological and structural characterization of M-NC. (a) XRD patterns of PS template, ZIF-8/PS precursor and M-NC-1000 samples. (b, c) SEM images and (d) TEM image of M-NC-1000. (e) N_2_ adsorption–desorption isotherms and pore size distributions of M-NC-x (x = 800, 900, 1000); (f) high resolution N 1s spectra of (1) M-NC-800, (2) M-NC-900, (3) M-NC-1000.

In this work, a PS template with a diameter range of 200–240 nm, as shown in the scanning electron microscopy (SEM) image in Fig. S1a,† was used to prepare the ZIF-8/PS (Fig. S1b†). The successful growth of ZIF-8 crystals in the voids of the PS template was proven by X-ray diffraction (XRD). The XRD pattern of ZIF-8/PS exhibits extra visible diffraction peaks compared with the PS template (Fig. 1a), which can be attributed to ZIF-8 crystals.^18^ Calcination of ZIF-8/PS at 1000 °C realizes the removal of the PS template and produces carbon as proven by the XRD patterns (Fig. 1a). SEM (Fig. 1b and c) and transmission electron microscopy (TEM, Fig. 1d) images reveal that the produced carbon is 2–3 microns, tetrakaidecahedron shape, and with a 3D ordered macroporous structure. The magnified FESEM image and TEM image of M-NC-1000 (Fig. 1c and d) illustrate the size of the macropores in the range 110 to 150 nm (Fig. S2b†), which is smaller than the PS template (200–240 nm, Fig. S2a†). This implies that the overall structure of ZIF-8/PS shrinks caused by the transformation of organic ligands into carbon and removal of the PS template at high temperature. To investigate the influence of temperature on the structural and physical properties, ZIF-8/PS was treated from 800 to 1000 °C, and the corresponding carbon is denoted as M-NC-x (x = 800, 900, 1000).

The N_2_ adsorption–desorption isotherms of M-NC-x (Fig. 1e) reveal isotherm characteristics of mixed type, implying the presence of wide pore ranges from micropore to macropore. The specific surface area and pore volume of M-NC-x is calculated and summarized in Table S1.† The higher temperature from 800 to 1000 °C helps to increase the specific surface area and pore volume from 252 to 497 m^2^ g^−1^, 0.253 to 1.074 cm^3^ g^−1^, respectively. This trend can be ascribed to the existence of micropores which is revealed by the higher nitrogen uptake at low relative pressure (*P*/*P*_0_ < 0.1) in the isotherms (Fig. 1e).

The elemental contents of M-NC-x, as summarised in Table 1, were investigated by X-ray photoelectron spectroscopy (XPS). The high-resolution C 1s spectra are fitted with five bands (Fig. S3a†), including sp^2^ carbon (284.5 eV), sp^3^ carbon (285.1 eV), C–O/C–N (286.0 eV), C=O (287.6 eV), and π–π* (∼290.5 eV), indicating the doping of oxygen and nitrogen in the carbon frameworks.^19,20^ As shown in Fig. 1f, the high-resolution N 1s spectra can be deconvoluted into four or two bands including pyridinic N (398.4 eV, N1), pyrrolic N (399.5 eV, N2), graphitic N (400.7 eV, N3), and oxidized N species (402–405 eV, N4).^21,22^ The nitrogen types and their percentages in M-NC-x are summarized in Table S2.† For M-NC-x, the ratios of pyridinic N and pyrrolic N are basically decreased, while the ratio of graphitic nitrogen increases when the carbonization temperature increases from 800 to 1000 °C due to the more stable graphitic N in the carbon frameworks.^23^ The nitrogen content in M-NC-x (x = 800, 900, 1000) is estimated to be 13.26, 8.54, and 3.73 at%, respectively. The decreased N content at a higher temperature is reasonable, considering the more extensive thermal treatment.

**Table 1:** The elemental content (atomic%) in M-NC-x (x = 800, 900, 1000) and M-PNC-y (y = 800, 900, 1000)

Samples	C	N	O	P	Zn
M-NC-800	80.60	13.26	5.04	—	1.1
M-NC-900	83.62	8.54	6.27	—	1.57
M-NC-1000	90.23	3.73	5.76	—	0.28
M-PNC-800	65.37	5.64	21.99	6.29	0.71
M-PNC-900	79.58	4.33	12.73	2.76	0.6
M-PNC-1000	86.17	3.17	9.54	1.12	—

### Characterization of M-PNC

**Fig. 2:**
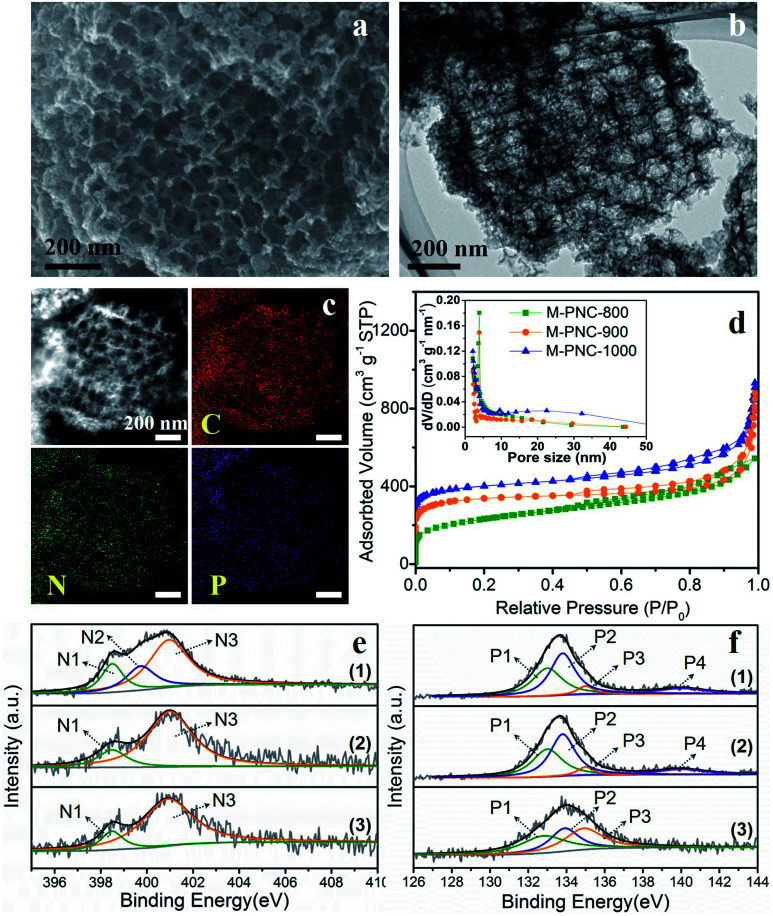
(a) SEM, (b) TEM, (c) STEM and elemental mapping images of M-PNC-1000. (d) N_2_ adsorption–desorption isotherms and pore size distributions of M-PNC-y (y = 800, 900, 1000); for clarity, the isotherms of M-PNC-900 and M-PNC-1000 were off-set by 70 and 140 cm^3^ g^−1^. High-resolution XPS spectra of (e) N 1s and (f) P 2p of (1) M-PNC-800, (2) M-PNC-900, (3) M-PNC-1000.

The as-prepared M-NC-x was post-doped with phosphorus by mixing with phytic acid (PA) and going through a secondary carbonization process at the same temperature as before (x = 800, 900, 1000). The selected ratio of PA to M-NC-x is 12 in this work, as determined by control experiments, and the results are discussed in the note for Fig. S4 in the ESI.† The macroporous phosphorus and nitrogen-doped carbons obtained were labelled as M-PNC-y (y = 800, 900, 1000). The morphology of the representative samples of M-PNC-1000 were characterized and are shown in Fig. 2a–c. M-PNC-1000 preserves the ordered macroporous structure, but the tetrahedral morphology was somewhat deformed. Also, M-PNC-1000 shows a much rougher surface after phosphorus doping which might occur due to the etching effect of PA as implied by the low yield of M-PNC when using excessive PA. The interconnected macroporous channels provide short pathways for reactant/product diffusion within the material, and the rough surface might give many more active sites. Elemental mapping of M-PNC-1000 shows the uniformly distributed carbon, nitrogen, and phosphorus (Fig. 2c).

N_2_ adsorption–desorption measurements were conducted to investigate the porosity of M-PNC-y. M-PNC-y (Fig. 2d) shows similar isotherms to M-NC-x (Fig. 1e), implying a similar hierarchical porous structure consisting of micropores, mesopores, and macropores. The specific surface area and pore volume of M-PNC-y is calculated and also summarized in Table S1.† The phosphorus doping has a significant impact on the porosity. For example, the resultant M-PNC-1000 (837 m^2^ g^−1^) shows a surface area almost 1.6 times as large as M-NC-1000 (497 m^2^ g^−1^). M-PNC-y displays a much higher nitrogen uptake at low relative pressure (P/P_*0*_ < 0.1) than M-NC-x (Fig. 1e and 2d), indicating the increased amount of micropores. Considering the process of phosphorus doping consists of a second carbonization treatment, it is unclear whether the increased porosity is caused by treatment with PA or the second carbonization treatment only. Thus, we carried out the second carbonization on M-NC-x without adding PA to produce M-NC-x-x; the second x represents the same temperature as the first time. The porosity values of M-NC-x-x were checked by N_2_ adsorption–desorption analysis as shown in Fig. S5a and Table S1.† The results show that the second treatment is in favor of increasing the porosity (mainly micropores as revealed by the N_2_ uptake at *P*/*P*_0_ < 0.1), especially at high temperatures of 900 and 1000 °C, while PA plays a more important role in increasing the porosity (mainly micropores as revealed by the N_2_ uptake at *P*/*P*_0_ < 0.1) at a relatively low temperature of 800 °C.

XPS investigated the main elemental compositions of M-PNC-y and M-NC-x-x. The states of carbon in M-PNC-y (Fig. S3b†) are similar to M-NC-x (Fig. S3a†). It is confirmed that the heat treatment plays a vital role in the content and state of nitrogen. Part of the less stable pyridinic N and pyrrolic N will be lost after the phosphorus doping or the second thermal treatment, leading to the decreased content of nitrogen (Table 1 and S3†). The ratio of pyridinic N and pyrrolic N in both M-PNC-y and M-NC-x-x (Fig. 2e, S5b and Table S2†) are reduced compared to the corresponding M-NC-x (Fig. 1f and Table S2†), especially at high temperatures. The oxygen content in M-PNC-y is much higher than M-NC-x and M-NC-x-x due to the treatment with phytic acid. The P concentration of M-PNC-y decreased from 6.29 at% to 2.76 and 1.12 at% upon increasing the calcination temperature from 800 to 1000 °C. As shown in Fig. 2f, the high-resolution P 2p XPS peaks located at *ca.* 133.0 eV (P1) and 134.0 eV (P2) can be attributed to P–C bonds and P–O bonds, respectively. The P in phosphate was located at 135 eV (P3) or higher binding energies (P4).

### Electrocatalytic properties of M-NC and M-PNC

**Fig. 3:**
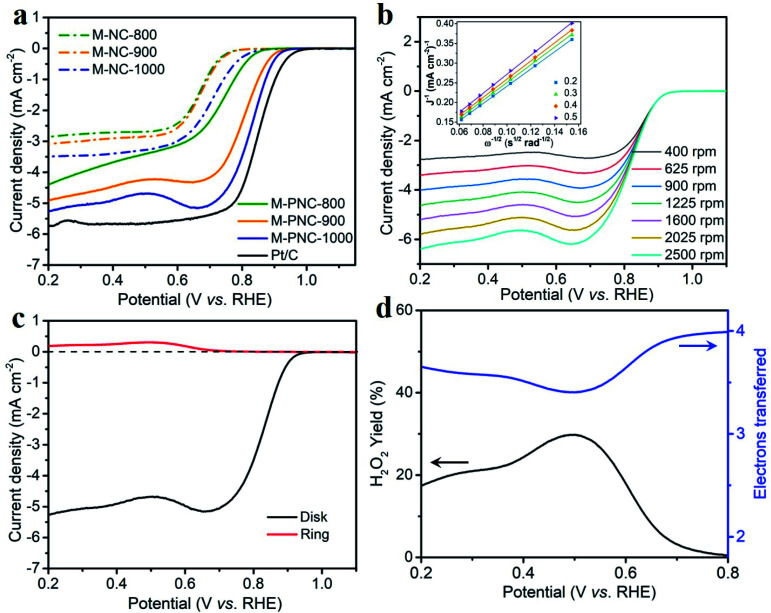
(a) LSV of M-NC-x and M-PNC-y (x, y = 800, 900, 1000), and Pt/C (20 wt%) at 1600 rpm with a scan rate of 10 mV s^−1^. (b) Different rotation speed, and (c) RRDE curves of M-PNC-1000 in O_2_ saturated 1 M KOH with a scan rate of 10 mV s^−1^; the inset in (b) is the Koutecky–Levich (K–L) plot. (d) Electron transfer number and peroxide yield of M-PNC-1000 calculated from (c).

Phosphorus doping might boost the catalytic activity of nitrogen-doped carbon materials for ORR. The electrocatalytic activities of M-NC-x, M-PNC-y, and Pt/C were evaluated using a rotating ring-disk electrode (RRDE) in alkali. As shown in the cyclic voltammetry (CV) curves obtained from an O_2_-saturated electrolyte (Fig. S6†), M-PNC-1000 displayed the most positive ORR peak at 0.821 V, compared to that of M-NC-x (0.652–0.684 V), M-PNC-800 (0.736 V), and M-PNC-900 (0.802 V). Linear sweep voltammetry (LSV) was commonly used to evaluate the catalytic performances for the ORR, and the scans are plotted in Fig. 3a. The onset potential (*E*_onset_) can be identified when the polarised current density achieves 0.20 mA cm^−2^ and is a significant value to appraise the performance of the ORR catalyst. M-PNC-y has a more positive *E*_onset_ compared with the corresponding M-NC-x. M-PNC-1000 shows the best catalytic activity with the most positive *E*_onset_ of 0.95 V, which is quite close to Pt/C (*E*_onset_ = 0.98 V), similar to M-PNC-900 (0.94 V), but much more positive than M-PNC-800 (0.89 V). Besides, M-PNC-1000 has a more positive half-wave potential (*E*_1/2_) of 0.84 V and diffusion-limited current density (*J*_d_) of 5.3 mA cm^−2^ compared with M-PNC-900 with *E*_1/2_ of 0.83 V and *J*_d_ of 4.9 mA cm^−2^. Above all, M-PNC-1000 shows the overall best performance for ORR amongst M-NC-x and M-PNC-y and becomes the top-ranked metal-free ORR electrocatalyst in alkali (Table S4†). The electron transfer number during ORR has been estimated using the Koutecky–Levich (K–L) plots derived from the LSV curves in Fig. 3b, and the average value is 3.8. It was also calculated from the RRDE LSV curves (Fig. 3c and d), which provide values 3.4 to 4.0 and the yield of H_2_O_2_ is 29% to 0%, respectively, from 0.5 V to 0.8 V (*vs.* RHE), implying that the M-PNC-1000 catalyst proceeds mainly *via* the four-electron dominant ORR path. Stability is another significant index for evaluating catalytic performance. CV cycling was performed on the most active M-PNC-1000 and Pt/C catalysts between 0.6 to 1.1 V for 5000 cycles. Fig. S7† shows the LSV curves of M-PNC-1000 and Pt/C catalysts before and after the accelerating test. M-PNC-1000 only displays 4% decay in diffusion limiting current density, a negative shift of *E*_onset_ = 10 mV and *E*_1/2_ = 30 mV. In contrast, the Pt/C catalyst shows 24% decay in the diffusion limiting current density, a negative shift of *E*_onset_ = 50 mV and *E*_1/2_ = 30 mV.

Although M-PNC-y catalysts possess a lower content of nitrogen than M-NC-x, they possess higher specific surface area and degree of graphitization, as well as additional doping of phosphorus. To clarify the function of phosphorus on ORR, we performed the electrochemical test on M-NC-x-x which has similar properties to M-PNC-y except for the P-doping. As shown in the Fig. S8,† the catalytic performance of M-NC-x-x for ORR is improved compared with M-NC-x due to the higher porosity and increased degree of graphitization after going through the second carbonization. However, M-PNC-y still displays much higher catalytic performance than M-NC-x-x, proving that P-doping plays an essential role in catalyzing ORR.

### Computational models of P–N doped carbon systems for ORR

Based on these results, the superior ORR performance of M-PNC-1000 is mostly attributed to the dual-doping of nitrogen and phosphorus. Thus, computational models of the P–N doped systems were designed to investigate the effect of phosphorus doping on nitrogen-doped carbon materials for ORR. In previous reports, it has been found that pyridinic N atoms in two-atom vacancies are highly thermodynamically stable as well as active towards the ORR.^24–26^ Though the effect of the graphitic-N and P co-doping has previously been investigated,^14*b*,27^ the impact of having both pyridinic and graphitic N atoms present is mostly unexplored. Furthermore, the influence that dopant symmetry has on the resulting catalytic behaviour has not yet been elucidated. Based on the characterization results, model surfaces based on pyridinic N, graphitic N and P were developed to test for the effect of P–N co-doping on ORR activity. Density functional theory calculations were undertaken to investigate the ORR, the details of which are contained in the ESI.†

**Fig. 4:**
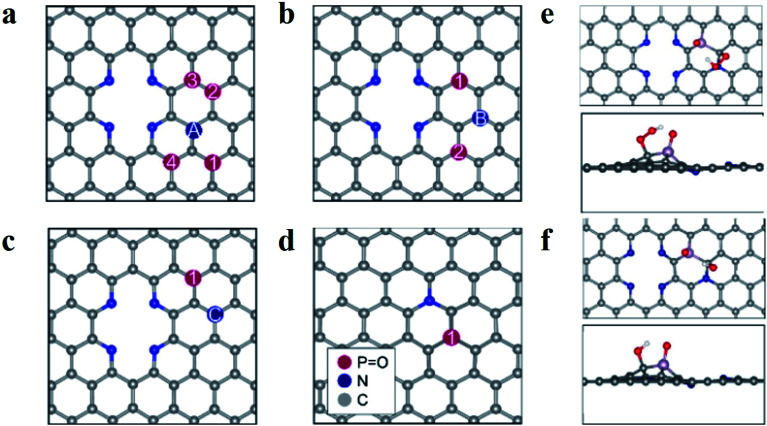
Schematic diagrams of surfaces and adsorption behaviour on M-PNC-1000. (a–d) Schematic diagrams of surfaces examined in this study; the pyridinic N atoms are the same in (a–c), and only the graphitic N is changing, and its position is denoted with letters; the P=O functionalized surface is shown with a single graphitic N in (d). (e and f) Illustrate the adsorption behaviour of the oxygen species OOH (e) and OH (f) to PNCB-1; the colours represent C in grey, N in blue, P in purple, O in red, and H in white in (e and f).

A schematic diagram summarising the systems is featured in Fig. 4a–d. For figures showing individual surfaces, see the ESI, Fig. S9.† In Fig. 4a–c, a two-atom vacancy in graphene is doped with four pyridinic N atoms and paired with a graphitic N, labelled A–C. The position of a phosphate group, P=O, was then changed (numbered in Fig. 4a–c) so that the P atom is situated at different distances from both the graphitic and pyridinic N atoms. We adopt the notation PNCX-Y where X is the letter corresponding to the position of the N atom, and Y is the position of the phosphate group. In each system, the P atom is close in proximity to at least one N atom and no further than two C atoms away, to maximize the local effects altering the ORR activity. The same systems without the P=O group (denoted NC-X) and a single P–N coupled surface (Fig. 4d) were also investigated. The P=O group was chosen based on the relative stability of P with a lone pair (P–R_3_), *versus* various forms of oxygenated P, where O is known to have the strongest interaction of all the intermediates in the ORR.^28^ It was found that the strongest interaction was with P=O and that the presence of O intermediates poisoned the surfaces as the binding energies were too large for the ORR to proceed. This is consistent with results demonstrating that the formation energy of P=O is lower than P-R_3_ and that the interactions of P-R_3_ are too significant for the ORR to proceed on the P atom. Hence the 4e^−^ mechanism of the ORR was investigated with the P=O interaction included for the PNCX-Y and P–N surfaces.

The study of the 4e^−^ mechanism revealed that in most cases, the sites that were the most active towards adsorption of the oxygen species were the carbon atoms surrounding the P=O group (see Fig. 4e and f). An exception is PNCA-3, which is the only case where the graphitic N is two C atoms away from the P group (see Fig. S9†). It is found that the carbon atom between the pyridinic N and graphitic N is the most active and results in an inactive system (see Table S5†). Where there was no P=O group, then adsorption was at the C atoms surrounding the graphitic N. If there was no graphitic N, adsorption was at the apex carbon in the pyridinic vacancy (see Fig. S9†). This was again consistent with other studies of P and N co-doping systems.^23^ In each case, the most active C atom is the carbon which is adjacent to the graphitic N atom. This suggests that N coupling makes the adjacent C atom more active towards adsorption compared to the other carbon atoms. The interactions of the ORR intermediates with the P=O group (see Fig. 4e and f) show that the H atom of the oxygen species of OOH and OH are consistently oriented towards the electronegative O atom of the phosphanone group. These results indicate that this stabilizing interaction is essential for the ORR to proceed.

The reaction profile for the 4e^−^ mechanism of the ORR is shown in Fig. S10.† The position of the phosphanone group appears to change the catalytic activity of the surfaces substantially, as the free energy of the OOH step, Δ*G*_OOH*_, ranges from 4.42 to 5.12 eV (see Table S5†). However, it is apparent that the best system is the NC-C system, as the magnitude of the O_2_ → OOH* step is smallest at the equilibrium potential with a magnitude of 0.73 eV. This finding is consistent with a computational report that carbon atoms next to graphitic N have a lower overpotential for the ORR than P=O within a two-carbon atom radius.^24^ However, it was found that this behaviour improved at further distances from P=O. Furthermore, it is worth noting that most comparable surfaces are those where the graphitic N is in the same position, *e.g.* PNCA-Y, where the graphitic N is in the A position though the P=O group is placed in different situations. These trends reveal that depending on the location of the P=O relative to graphitic N, the performance can be improved relative to the ORR with the four-atom vacancy only, and P–N, which is comprised of the single P=O and graphitic N.

The change in the active site for NC-D to the vacancy apex (see Fig. S9†) indicates that graphitic N must be incorporated for the surface without the phosphoryl group to be sufficiently active for the ORR. This is because the free energy for adsorption for the NC-D case for the OOH step is positive and significantly disfavoured compared to the other studied systems. However, the calculated magnitudes show that the NC-D system is incapable of catalyzing the ORR. Other reports have found the same active site with different methods.^22^ Therefore, the values in this work should be treated as relative values, and the systems displaying better performance based on the magnitudes should be reasonably expected to perform well in experiments.

Based on the calculated overpotentials (see Table S5†), NC-A and NC-B perform worse than the corresponding phosphorus-doped systems (PNCA-2 and PNCB-1) by 0.23 V and 0.04 V, respectively. This suggests that adding P=O to these systems can enhance the catalytic activity that is observed in PNC catalysts. Furthermore, it appears that the P–N system (see Fig. 4d) without the pyridinic nitrogen, performs worse than most of the surfaces, indicating that an enhancement occurs when a vacancy is adjacent to a P–N site. Comparing PNCC-1 and NC-C, this apparent improvement is due to the presence of graphitic N, as the activity of PNCC-1 is worse than NC-C. However, comparing the NC-A, NC-B and P–N surfaces, it appears that the PNCX-Y structures improve both the performance of the systems in the absence of the P=O as well as the pyridinic N atoms. Thus, the presence of mixed vacancy or N types may drastically change the performance based on their relative positions only.

## Conclusion

In summary, a face-centred cubic PS template was used to grow the single-crystal ZIF-8 precursors (ZIF-8/PS) by double-solvent-induced methods. After calcination and post-doping, ordered macroporous phosphorus and nitrogen-doped carbon (M-PNC) was prepared successfully. Thanks to the overall hierarchical porous structural and compositional advantages, M-PNC-1000 exhibits an unusual catalytic activity for ORR in an alkaline electrolyte, which is much higher than M-NC and is comparable to current heteroatom-doped carbon-based catalysts for ORR. Density functional theory calculations revealed that N coupling makes adjacent C atoms more active towards adsorption compared to the other carbon atoms. With a four-atom vacancy, the ORR performance can be improved depending on the location of the P=O relative to graphitic N. This supports how the inclusion of dopants like phosphorus can enhance the performance of electrocatalysts for ORR.

## Conflicts of interest

There are no conflicts to declare.

## Supplementary Material

SC-011-D0SC02518F-s001
