# Supplementary material for: Highly ordered macroporous dual-element-doped carbon from metal–organic frameworks for catalyzing oxygen reduction
Source: Chem Sci. 2020 Aug 11;11(35):9584–92. doi: 10.1039/d0sc02518f (PMC8162149; doi:10.1039/d0sc02518f)
Supplement: SC-011-D0SC02518F-s001 [file SC-011-D0SC02518F-s001.pdf]

Electronic Supplementary Material (ESI) for Chemical Science.
This journal is © The Royal Society of Chemistry 2020

Supporting Information

1. Experimental Section

1.1 Chemicals: Ammonium persulfate ($(NH_4)_2S_2O_8$, 99%, Sinopharm Chemical Reagent Co., Ltd.), styrene (99%, Aladdin Industrial Corporation), polyvinyl pyrrolidone (PVP, K30, Sinopharm Chemical Reagent Co., Ltd.), potassium hydroxide pellets (KOH, 99%, Sinopharm Chemical Reagent Co., Ltd.), zinc nitrate hexahydrate ($Zn(NO_3)_2{\cdot}6H_2O$, 99%, Sinopharm Chemical ReagentCo., Ltd.), 2-methylimidazole (98%, Aladdin Industrial Corporation), phytic acid (50%, Macklin), methanol (CH_3OH,99%, Sinopharm Chemical Reagent Co., Ltd.), ethyl alcohol (C_2H_5OH, Sinopharm Chemical Reagent Co., Ltd.). All the chemicals used in this work were of analytical grade and were used without further purification.

1.2 Synthesis of polystyrene sphere cakes: The polystyrene sphere (PS) cakes were prepared according to the literature.[S1] In detail, 6 mL of styrene was purified by using a 10 wt% KOH solution and was added to a three-necked round-bottomed flask. Then, 50 mL water consisted of 250 mg of PVP K30 was added into the above solution. After bubbling using argon for 30 min, the mixture was fluxed at 70 °C under magnetic stirring. After stirring for another 30 min, 10 mL of aqueous solution containing 0.15 g $(NH_4)_2S_2O_8$ was quickly injected to initiate the polymerization reaction of styrene. The mixture turned to milk-like liquid after stirring for 20 h at 70 °C in a water bath. Finally, the polystyrene aqueous solution was directly filtered under reduced pressure to obtain polystyrene cakes. During the vacuum filtration, the white filter cake layer was washed

by water and ethyl alcohol to remove the absorbed ions and organic molecules. After that, the white polystyrene sphere cakes were dried at 60 °C overnight.

1.3 Synthesis of ZIF-8/PS precursor: The ZIF-8/PS precursor was synthesized via a confinement crystal growth method. The white PS cakes were immersed into a ZIF-8 precursor solution of 8.15 g $Zn(NO_3)_2 \cdot 6H_2O$, 6.75 g 2-methylimidazole, and 45 mL methanol in a 100 mL beaker for 1 h. After that, the beaker was further degassed in a 25 °C vacuum oven for 15 min to make all spacing in PS cakes were filled by precursor solution. The impregnate PS cakes were moved to a clean bottle and dried at 60 °C for 10 h. The precursor-contained PS cakes were soaked in a $CH_3OH/NH_3{\cdot}H_2O$ (1:1 v/v) solution at a room temperature and were treated with vacuum degassing for 5 min to guarantee the adequate permeation of solvent into the polystyrene cakes. After that, the mixture was placed at the room temperature and atmospheric pressure for 24 h. The ZIF-8/PS precursor was then filtrated, washed by methanol, and dried in air.

1.4 Preparation of ordered macroporous nitrogen-doped carbon (M-NC): The ordered macroporous nitrogen-doped carbon (M-NC) was synthesized by directly calcining the ZIF-8/PS precursor in a nitrogen atmosphere with a heating rate of 10 °C /min, then were kept at the target temperature for 2 h. The final calcination temperatures were 800 °C, 900 °C, and 1000 °C, respectively. The obtained samples were named as M-NC-*x* (x = 800, 900, 1000). M-NC-*x* samples were carbonized again at the same final temperature to produce M-NC-*x*-*x* (x = 800, 900, 1000).

1.5 Synthesis of ordered macroporous phosphorous and nitrogen-doped carbon (M-PNC): In a typical synthesis, 10 mg as-prepared M-NC-*x* and 120 mg phytic acid

were mixed in 400 uL C_2H_5OH/H_2O (1:2 v/v) under sonication for 0.5 h. Afterward, the black liquid was transferred to a clean porcelain boat and dried in a 60 °C vacuum oven. Thus, M-PNC-*y* (*y* = 800, 900, 1000) was obtained by calcining the mixture of M-NC-*x* and phytic acid at temperatures equals to *x* (*x* = 800, 900, 1000) in nitrogen atmosphere. In control experiments, M-NC-1000 was treated with phytic acid of 30, 60, and 150 mg, producing M-PNC-1000-1, M-PNC-1000-2, M-PNC-1000-4, respectively. M-PNC-1000 prepared using the typical recipe (120 mg of PA) was also labeled as M-PNC-1000-3.

2. Physical characterization

The morphology of the as-synthesized materials was observed on a field emission scanning electron microscope (FESEM, Hitachi SU-8000) with an accelerating voltage of 10.0 kV. Transmission electron microscopy (TEM), scanning transmission electron microscopy (STEM) images, energy-dispersive X-ray spectroscopy (EDXS), and elemental mapping analysis were performed using a JEM-2100F (JEOL, Japan) operated at 200 kV. The nitrogen adsorption-desorption isotherms were obtained by using a BELSORP-mini (BEL, Japan) at 77 K. The specific surface area was evaluated by the multipoint Brunauer-Emmett-Teller (BET) method at a relative pressure from 0.05 to 0.3 based on the adsorption data. A Rigaku Rint 2000 X-ray diffractometer measured wide-angle X-ray diffraction (XRD) patterns with monochromatic Cu Kα radiation (40 kV, 40 mA) at a scanning rate of 2 ° min^{-1}. X-ray photoelectronic spectroscopy (XPS) spectrums were collected on a PHI Quantera SXM (ULVAC-PHI) instrument with an Al Kα radiation, and the binding energies were calibrated by

referencing them to the C 1s (284.5 eV) binding energy.

3. Electrochemical measurements

The electrochemical measurements of oxygen reduction reaction (ORR) were conducted on a CHI842B electrochemical workstation with a standard three-electrode electrochemical cell. Pt wire and saturated calomel electrodes (SCE) were selected as the counter electrode and reference electrode, respectively. The working electrode was prepared by dropping catalyst inks onto the rotating ring-disk electrode (RRDE) electrode. In the typical experiment, 5 mg of catalyst and 50 uL of 5.0 wt % Nafion solution was dispersed in 950 μL of isopropanol/water (1:2 v/v) mixture. The suspension was further sonicated for 1 h to obtain a homogeneous ink. Then 5 μL of the black ink was dropped on a 4 mm diameter RRDE electrode. O_2/N_2 saturated 0.1 M KOH aqueous solutions were selected as the electrolytes, and the flow of O_2/N_2 was maintained during tests. Cyclic voltammetry (CV) and linear sweep voltammetry (LSV) measurements were conducted with the scan rates of 50 and 10 mV s^{-1}, respectively. The rotating speeds for RRDE electrode were 400, 625, 900, 1225, 1600, 2025 and 2500 rpm, while the potential for a ring electrode was held at 1.2 V vs. RHE. The current densities were estimated based on the geometric area of the working electrode and the potential values were calibrated to the reversible hydrogen potential (E_{RHE}) based on the Nernst equation of $E_{RHE}=E_{SCE}+0.241+0.0591*pH$.

The H_2O_2 yield (% H_2O_2) and electron transfer number (n) was calculated as the following equations:

$$\%H_2O_2 = 2 \times \frac{I_R/N}{I_D + I_R/N} \times 100\% \quad (1)$$

$$n = 4 \times \frac{I_D}{I_D + I_R/N} \quad (2)$$

Where I_D is disk current and I_R is ring current, and N is the collection efficiency of the ring electrode (0.37 in this work).

The Koutecky-Levich equation is：

$$J^{-1} = J_k^{-1} + (B\omega^{0.5})^{-1} \quad (3)$$

$$B = 0.2nF(D_{O_2})^{2/3}\tau^{-1/6}C_{O_2} \quad (4)$$

in the equation, J_K represents kinetic current density, which is calculated from J and J_d ($J_k = (J*J_d)/(J_d–J)$), where J is the current density at 0.72 V and J_d is limited current density. ω means rotating rate of electrode, n indicates the electrons transferred number per oxygen molecule, F is the Faraday constant (96485 C mol^{-1}), D_{O2} stands for the diffusion coefficient of O_2 in the electrolyte (1.9×10^{-5} cm^2 s^{-1}), τ represents kinetic viscosity of the electrolyte (0.01 cm^2 s^{-1}), and C_{O2} denotes the saturated concentration of O_2 (1.2×10^{-6} mol cm^{-3}) in aqueous system. The constant 0.2 is used to determine B when the unit of rotation speed is rpm.

4. Computational Models of P-N doped carbon systems for ORR

4.1 Oxygen reduction reaction mechanisms: There are two ways to reduce O_2, the $4e^-$ and $2e^-$ mechanisms. The $4e^-$ pathway reduces O_2 completely to $2H_2O$ which is desirable for a fuel cell:

$$O_2 + 4(e^- + H^+) \rightarrow 2H_2O \; E_0 = 1.23 \text{ V}$$

In this mechanism, there are two means to generate water, notably the associative and dissociative pathways, which produces either *OOH from the addition of hydrogen to O_2 or 2O* via the dissociation of O_2, respectively. However, since it is known that the experimental catalysts in this study selectively produce H_2O, it is practical to optimize the 4e$^-$ mechanism based on the relevant thermodynamically derived binding energies. The 4e$^-$ associative mechanism is as follows:

$$O_2 + e^- + H^+ \rightarrow {}^*OOH$$

$$^*OOH + e^- + H^+ \rightarrow {}^*O + H_2O$$

$$^*O + e^- + H^+ \rightarrow {}^*OH$$

$$^*OH + e^- + H^+ \rightarrow H_2O.$$

4.2 Computational methods: The calculations were carried out within the framework of density functional theory (DFT).[S2] Exchange-correlation was treated using the revised Perdew, Burke, and Ernzerhof functional (RPBE).[S3] Dispersion forces were processed using the D3 correction of Grimme.[S4] A periodically-projected plane-wave basis set was employed using the projector-augmented wave (PAW) method with a kinetic energy cutoff 450 eV.[S5] The spin-polarisation was considered for the treatment of the oxygenated species. The models were developed based on an 8 x 4 graphene cell, with lattice parameters of 19.74 x 17.04 and a 20 Å vacuum above the graphene layer. For visualization of coordinate data, VESTA was used.[S6]

4.3 Calculating free energy values and computational hydrogen electrode model: The computational hydrogen electrode model was adopted in the manner of Nørskov et

al.[S7] This equates the free energy of each electron-proton pair as equal to half that of gaseous hydrogen at equilibrium, hence using the reversible hydrogen electrode as the reference potential. The effect of the potential on the free energy changes in the mechanism is defined as the following:

$$\Delta G = \Delta E - \Delta ZPE - T\Delta S - \Delta G_{pH} - neU \text{ ,} \quad ()$$

where ΔE is the DFT-calculated reaction energy, ΔZPE is the difference in the zero-point energy of the reactants and products, $T\Delta S$, the entropic contribution to the reaction free energy, ΔG_{pH} is the effect of the pH on the potential and neU describes the potential eU relative to the reversible hydrogen electrode scaled by the number of electrons, n. In our study, we add zero-point energy and entropy contributions to the calculated ΔE to define the free energies of each ORR intermediate, and these are given in below table.

The DFT calculated energies of O_2 and O-containing species, such as the peroxy intermediates, are known to exhibit significant calculation errors. This is due to limitations of DFT's capacity to correctly describe the ground state of O_2. Following Man et al.,[S8] in our approach, we limit systematic errors by using the energies of H_2 and H_2O as reference energies and refer to the experimental value of the fuel cell reaction of 4.92 eV. Furthermore, as all the steps in the ORR that produce H_2O can result in either an adsorbed or bound water, we exclusively calculate the free water case. Additionally, we do not specifically carry out calculations with H_2O above the surface as it is not expected to significantly contribute to the energy of the intermediate on the surface and hence the potential barrier dependence of the intermediates for our systems. For example, the reaction energy for *OOH was calculated as follows:

$$\Delta E_{*OOH} = E_{*OOH+SF} - E_{SF} - (2E_{H_2O} - \frac{3}{2}E_{H_2})$$ ()

A similar analysis was used for *OH and *O. In all cases, multiple initial geometries of the adsorbate were tested for their interaction with the surface. The most stable geometries were used. The combined ZPE and entropy corrections for the adsorbed species at 298 K are shown in the following table and were added for each of the intermediates: [S9]

**Intermediate**	**ΔZPE-TΔS（eV）**
*OOH	0.40
*OH	0.35
*O	0.02

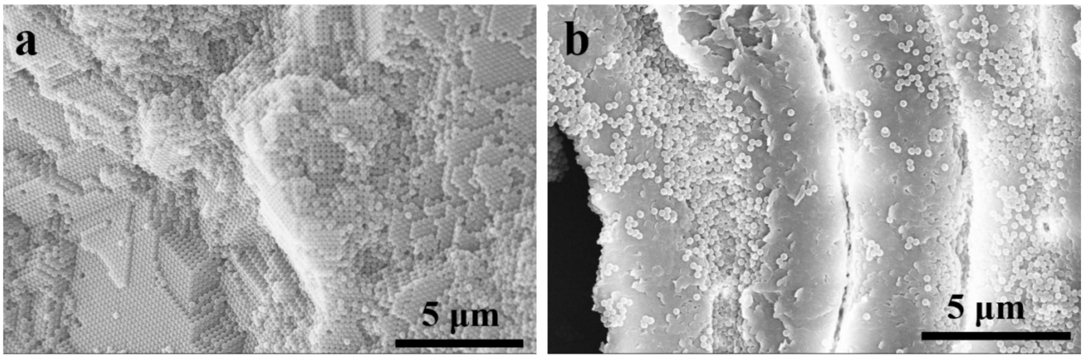

Figure S1. SEM images of (a) PS cake and (b) ZIF-8/PS.

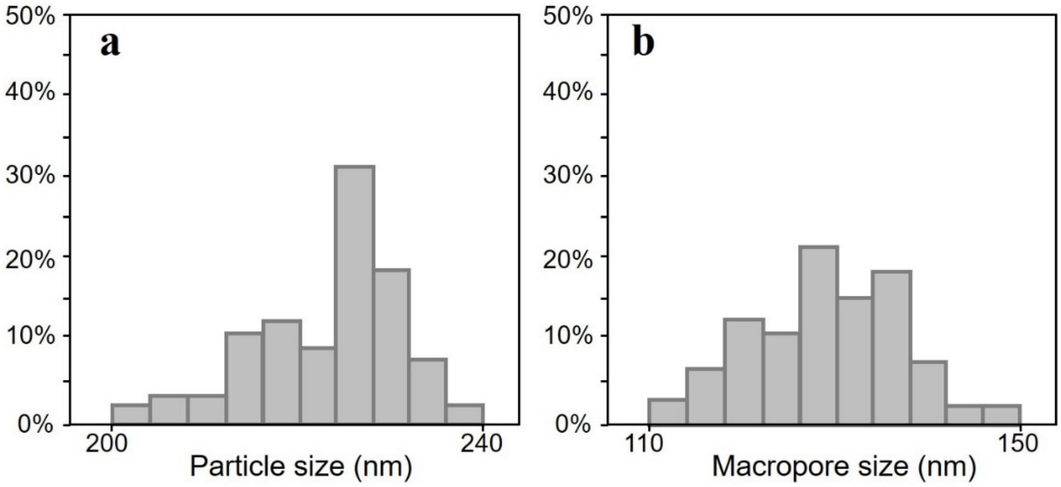

Figure S2. (a) Particle size distribution histograms that calculated from over 50 particles of PS spheres in Figure S1a and (b) the pore size distribution histograms that calculated from Figure 1c.

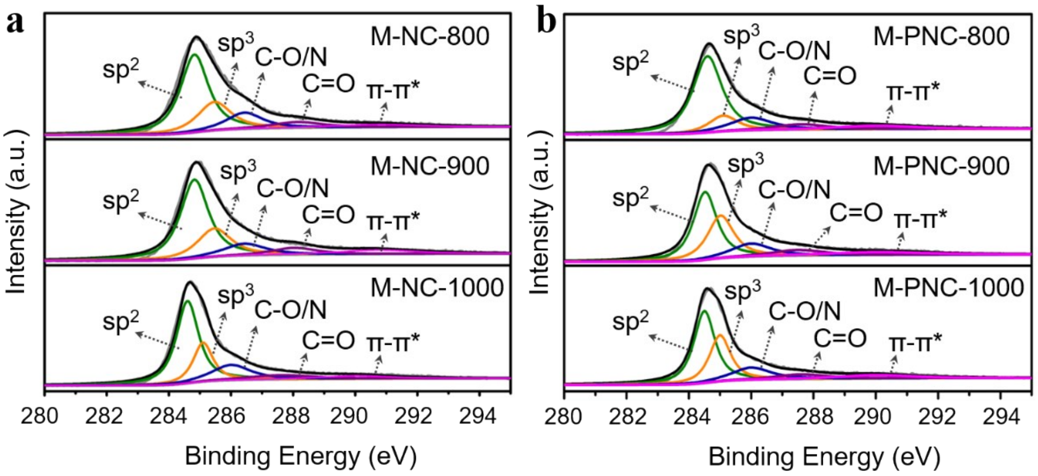

Figure S3. High resolution C 1s spectra of (a) M-NC-*x* (*x* = 800, 900, 1000) and (b) M-PNC-*y* (*y* = 800, 900, 1000).

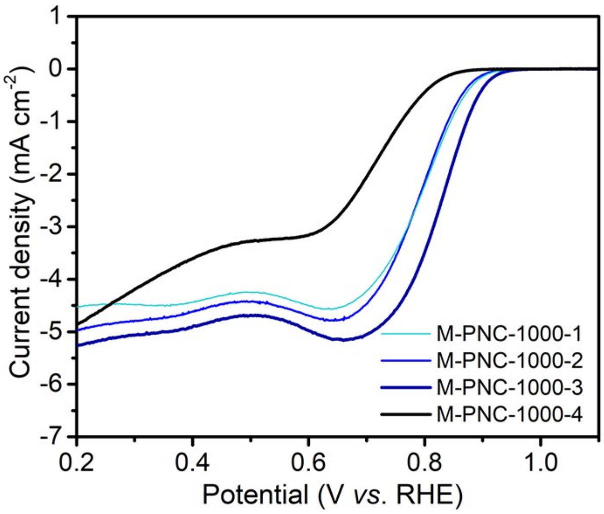

Figure S4. LSV of M-PNC-1000-1,2,3,4 in O_2 saturated 0.1 M KOH with a scan rate of 10 mV s^{-1} at a rotation speed of 1600 rpm.

Note for Figure S4: Different amounts of phytic acid (PA) were used in the control experiments to determine the suitable ratio of PA to M-NC. PA of 30, 60, 120, and 150 mg were used to doping 10 mg of the representative M-NC-1000, producing M-PNC-1000-1, M-PNC-1000-2, M-PNC-1000-3, and M-PNC-1000-4, respectively. The electrochemical testing shows that M-PNC-1000-3 shows the best performance (Figure S4), thus we use this ratio as the typical recipe throughout the work. It is worth to mention that the excessive use of PA will lead to a low yield of M-PNC due to the etching effect under second carbonization, resulting in a worse catalytic performance.

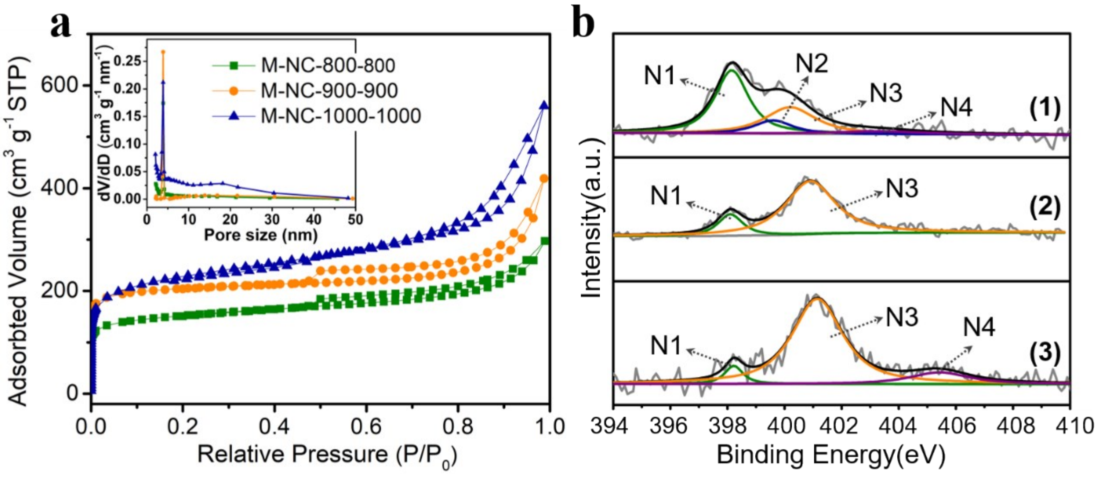

Figure S5. (a) Nitrogen adsorption-desorption isotherms and pore size distributions of M-NC-*x*-*x* (*x* = 800, 900, 1000). (b) High resolution XPS spectra of N1s for (1) M-NC-800-800, (2) M-NC-900-900, (3) M-NC-1000-1000.

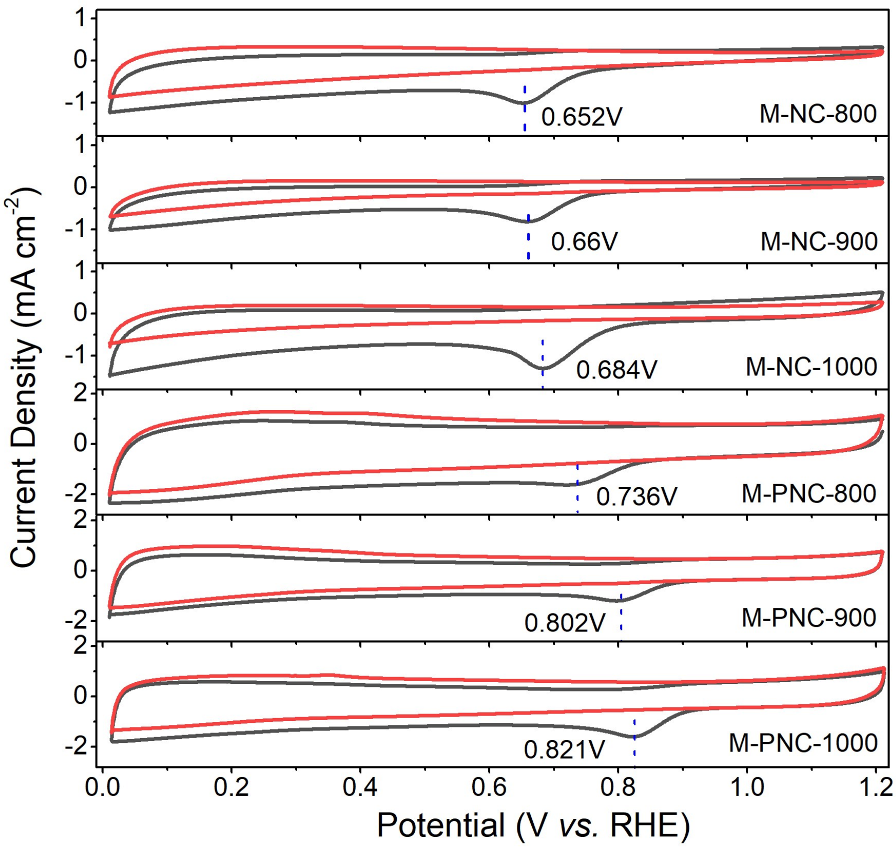

Figure S6. CV of M-NC-*x* and M-PNC-*y* (*x*, *y* = 800, 900, 1000) in N_2 (red line) and O_2 (black line) saturated 0.1 M KOH with a scan rate of 50 mV s^{-1}.

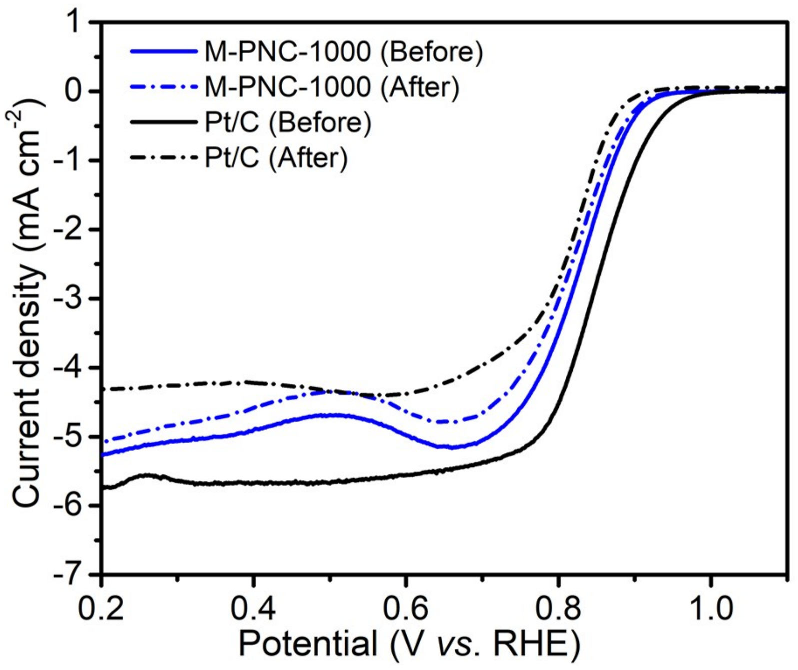

Figure S7. LSV of M-PNC-1000 and Pt/C before and after stability test in O_2 saturated 0.1 M KOH with a scan rate of 10 mV s^{-1} at a rotation speed of 1600 rpm.

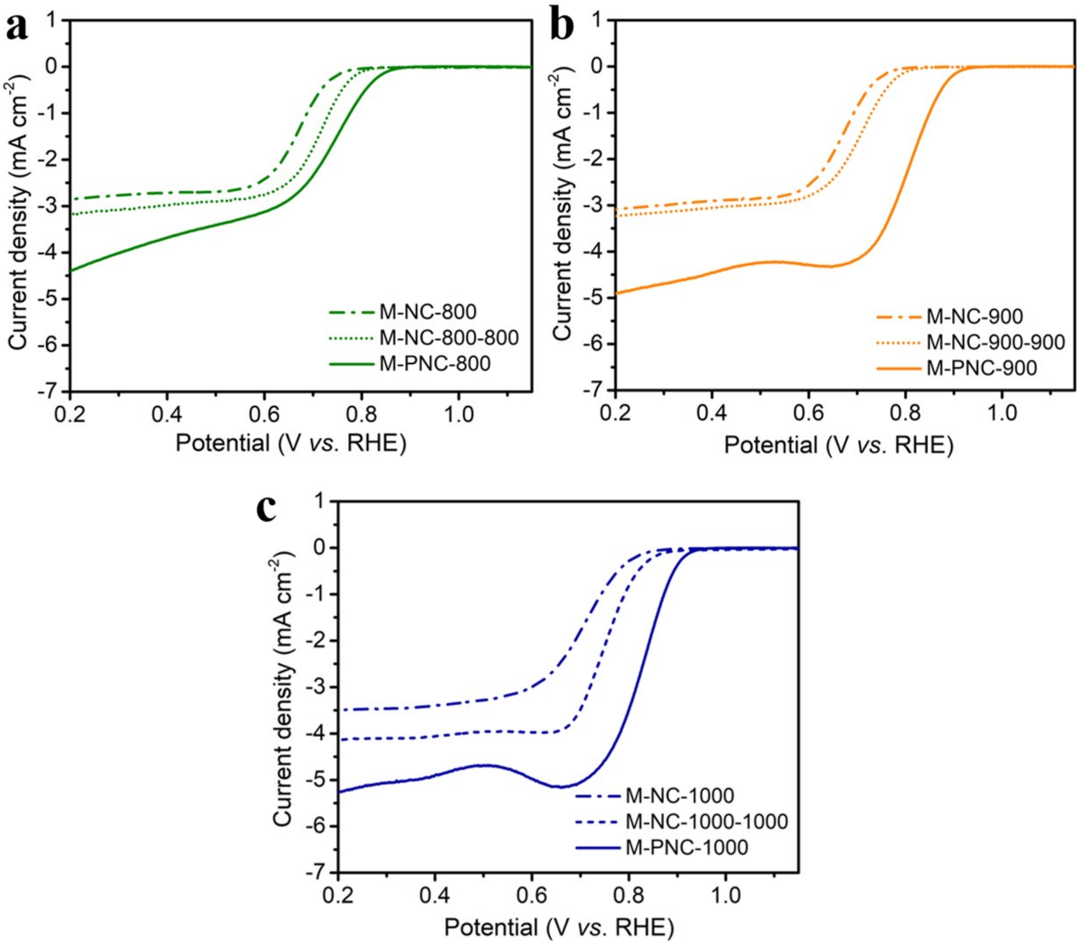

Figure S8. LSV of M-NC-*x*, M-NC-*x*-*x*, and M-PNC-*y* (*x*, *y* = 800, 900, 1000) in O_2 saturated 0.1 M KOH with a scan rate of 10 mV s^{-1} at a rotation speed of 1600 rpm.

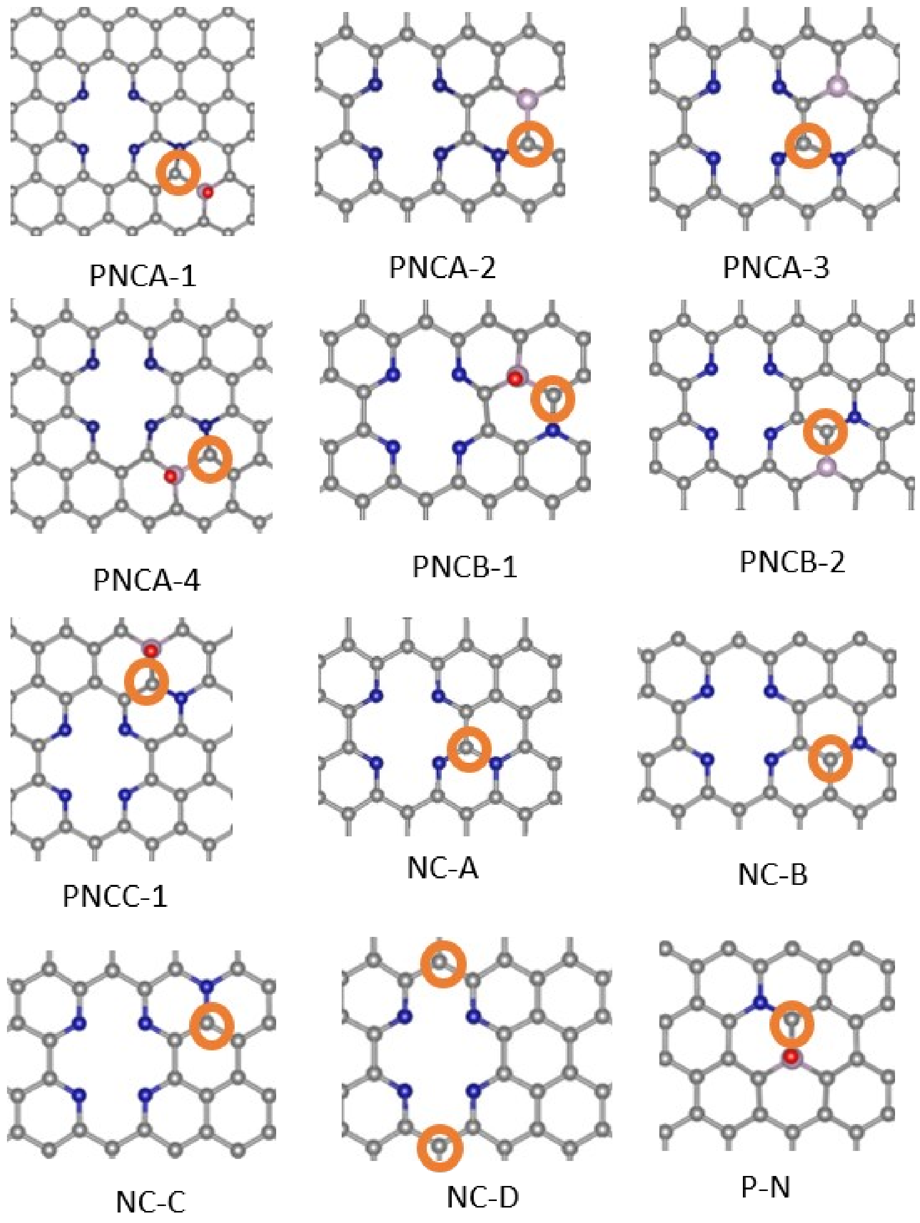

Figure S9. Active sites shown in orange for the ORR for the investigated models. The colours represent C in grey, N in blue, P in purple, and O in red.

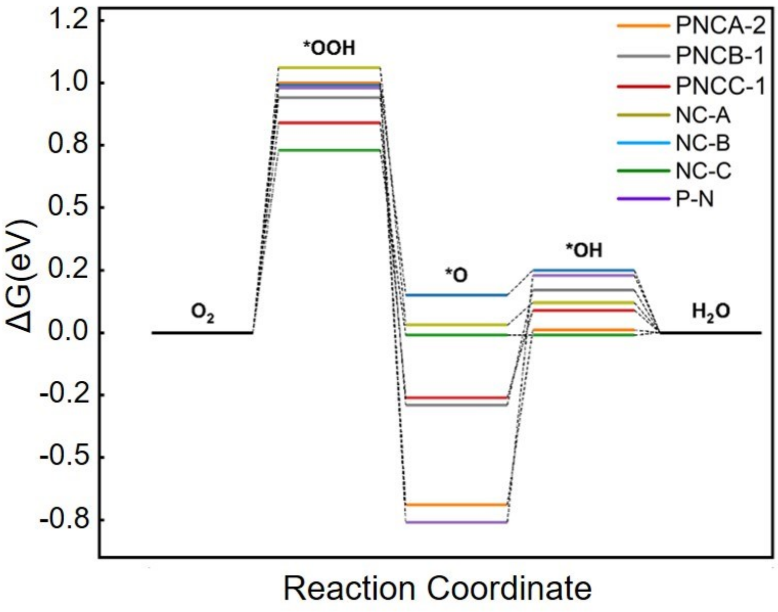

Figure S10. Free energy profile of best performing surfaces for the 4e- mechanism of the ORR. The profile is shown at the equilibrium potential, U_0 = 1.23 V. The positions for the free energy of adsorption of OOH on the surfaces PNCA-2, P-N and PNCC-1 are offset by 0.01 eV for clarity.

Note for Figure S10: The reaction profile for the 4e- mechanism of the ORR is shown in **Figure S10** at the equilibrium potential U_0 = 1.23 V. Only the results for the best performing surfaces are shown. The free energy values at U = 0 and the calculated overpotentials are tabulated in **Table S5**. From the reaction profile, it is revealed that the thermodynamically limiting step is OOH adsorption, which is the step which is largest at U_0. This behaviour shows that the PNC co-doped species weakly bind oxygen, which is typical for non-metal-based catalysts.

Table S1. The specific surface area (S_{BET}) and pore volume (V_{pore}) of M-NC-*x* and M-NC-*x*-*x* (*x* = 800, 900, 1000), and M-PNC-*y* (*y* = 800, 900, 1000).

**Samples**	**S_{BET} (m^2 g^{-1})**	**V_{pore} (cm^3 g^{-1})**
M-NC-800	252	0.253
M-NC-900	442	0.605
M-NC-1000	497	1.074
M-NC-800-800	542	0.460
M-NC-900-900	731	0.648
M-NC-1000-1000	921	1.136
M-PNC-800	834	0.841
M-PNC-900	840	1.225
M-PNC-1000	837	1.211

Table S2. The types of nitrogen and their percentages doped in M-NC-*x* and M-NC-*x*-*x* (*x* = 800, 900, 1000), and M-PNC-*y* (*y* = 800, 900, 1000).

**Samples**	**N1 (%)**	**N2(%)**	**N3(%)**	**N4(%)**
M-NC-800	42.1	7.7	43.6	6.6
M-NC-900	41.9	9.4	44.1	4.6
M-NC-1000	15.9	/	84.1	/
M-NC-800-800	47.0	7.8	39.0	6.2
M-NC-900-900	17.0	/	83.0	/
M-NC-1000-1000	6.0	/	81.0	13.0
M-PNC-800	19.4	21.9	58.7	/
M-PNC-900	16.9	/	83.1	/
M-PNC-1000	14.9	/	85.1	/

Table S3. The elemental content (atomic%) in M-NC-*x*-*x* (*x* = 800, 900, 1000).

Samples	C	N	O	Zn
M-NC-800-800	85.93	8.80	4.81	0.46
M-NC-900-900	90.88	4.62	4.32	0.18
M-NC-1000-1000	92.18	3.18	4.48	0.16

Table S4. Comparison of the ORR performance of the recently reported carbon-based catalysts.

Catalyst	0.1 M KOH Electrolyte		
	E_{onset} (V vs. RHE)	$E_{1/2}$ (V vs. RHE)	$\|J_d\|$ (mA cm^{-2})
20 wt % Pt/C	0.98	0.86	6.2
meso/micro-PoPD[S10]	0.92	0.87	5.8
N,P-CGHNs[S11]	0.94	0.82	5.9
2D-PPCNs[S12]	0.92	0.85	4.5
NPMC[S13]	0.94	0.85	4.0
N,P-MC[S14]	0.95	0.84	5.0
N,P-NC[S15]	0.97	0.83	5.5
N/P/O-doped GP[S16]	0.95	0.79	5.4
M-PNC (This Work)	0.95	0.84	5.3

Table S5. Free energy profile for the 4e⁻ mechanism of the oxygen reduction reaction at U=0 V. The free energy values are displayed in eV. U_{onset} and the overpotential (ŋ) are in V. For the systems PONC-A3 and NC-D, the values are not displayed as at the reaction is non-spontaneous at a potential of 0 since the ΔG_{*OOH} > 4.92 eV.

Surface	ΔG_{*OOH}	ΔG_{*O}	ΔG_{*OH}	U_{onset}	η
PNCA-1	4.68	1.67	1.43	0.24	0.99
PNCA-2	4.52	1.77	1.24	0.40	0.83
PNCA-3	5.09	2.66	1.89	-	-
PNCA-4	4.68	1.56	1.44	0.12	1.11
PNCB-1	4.63	2.17	1.40	0.29	0.94
PNCB-2	4.67	1.84	1.42	0.25	0.98
PNCC-1	4.53	2.20	1.32	0.39	0.84
NC-A	4.75	2.49	1.35	0.17	1.06
NC-B	4.68	2.61	1.48	0.24	0.99
NC-C	4.42	2.45	1.22	0.50	0.73
NC-D	5.12	2.87	1.90	-	-
P-N	4.68	1.70	1.46	0.24	0.99

References:

[S1] K. Shen, L. Zhang, X. Chen, L. Liu, D. Zhang, Y. Han, J. Chen, J. Long, R. Luque, Y. Li and B. Chen, *Science*, 2018, **359**, 206–210.

[S2] G. Kresse and J. Furthmüller, *Phys. Rev. B*, 1996, **54**, 11169–11186.

[S3] B. Hammer, L. B. Hansen and J. K. Nørskov, *Phys. Rev. B*, 1999, **59**, 7413–7421.

[S4] S. Grimme, J. Antony, S. Ehrlich and H. Krieg, *J. Chem. Phys.*, 2010, **132**, 154104.

[S5] G. Kresse and D. Joubert, *Phys. Rev. B*, 1999, **59**, 1758–1775.

[S6] K. Momma and F. Izumi, *J. Appl. Cryst.*, 2011, **44**, 1272–1276.

[S7] J. K. Nørskov, J. Rossmeisl, A. Logadottir, L. Lindqvist, J. R. Kitchin, T. Bligaard and H. Jónsson, *J. Phys. Chem. B*, 2004, **108**, 17886–17892.

[S8] I. C. Man, H. Y. Su, F. Calle-Vallejo, H. A. Hansen, J. I. Martínez, N. G. Inoglu, J. Kitchin, T. F. Jaramillo, J. K. Nørskov and J. Rossmeisl, *ChemCatChem*, 2011, **3**, 1159–1165.

[S9] J. M. T. A. Fischer, Computational Studies of Approaches to Enhance the Catalytic Performances of Graphene and Graphitic Carbon-Nitride Materials, 2018, PhD thesis.

[S10] H. W. Liang, X. Zhuang, S. Brüller, X. Feng and K. Müllen, *Nat. Commun.*, 2014, **5**, 4973.

[S11] J. Yang, H. Sun, H. Liang, H. Ji, L. Song, C. Gao and H. Xu, *Adv. Mater.*, 2016, **28**, 4606-4613.

[S12] W. Lei, Y.-P Deng, G. Li, Z. P. Cano, X. Wang, D. Luo, Y. Liu, D. Wang and Z. Chen, *ACS Catal.*, 2018, **8**, 2464-2472.

[S13] J. Zhang, Z. Zhao, Z. Xia and L. Dai, *Nat. Nanotech.*, 2015, **10**, 444–452

[S14] Z. Zhang, J. Sun, M. Dou, J. Ji and F. Wang, *ACS Appl. Mater. Inter.,* 2017, **9**, 16236-16242.

[S15] H. Luo, W.-J. Jiang, Y. Zhang, S. Niu, T. Tang, L.-B. Huang, Y.-Y. Chen, Z. Wei and J.-S. Hu. *Carbon*, 2018, **128**, 97-105.

[S16] B. Huang, Y. Liu, X. Huang and Z. Xie, *J. Mater. Chem. A*, 2018, **6**, 22277-22286.
